# A case report of differentiated thyroid cancer presenting as a renal mass

**DOI:** 10.22038/AOJNMB.2021.60302.1422

**Published:** 2022

**Authors:** Javaid Iqbal, Asif Jamal, Basit Iqbal

**Affiliations:** 1 Karachi Institute of Radiotherapy & Nuclear Medicine, Karachi, Pakistan; 2 Liaquat National Hospital, Stadium Road, Karachi, Pakistan; 3 Sindh Institute of Urology & Transplant, Chand Bibi Road, Karachi, Pakistan; 4 Gujranwala Institute of Nuclear Medicine & Radiotherapy, Nizampur, Gujranwala, Pakistan

**Keywords:** Differentiated thyroid cancer, Papillary thyroid cancer, Renal metastasis

## Abstract

The kidney is an unconventional site for thyroid metastasis. As of the writing of this article, only about 30 cases have been reported. It presents like a renal mass. We are reporting a man with thyroid carcinoma presenting with distant metastasis to the kidney. He had complaints of abdominal pain and haematuria. Initial imaging suggested a left renal mass. A diagnosis of renal cell carcinoma was made and a nephrectomy was performed. Histopathology revealed it to be a metastasis from cancer of the thyroid gland. Subsequently, an ultrasound of the thyroid gland was performed, which showed a malignant appearing thyroid nodule. Correlative bone scan showed uptake at multiple skeletal sites. Total thyroidectomy was done and it was found to be papillary thyroid cancer. Subsequently, high dose radioactive iodine was administered. The patient was followed up and has recently found to have metastasis to the brain and is undergoing radiotherapy.

## Introduction

 Differentiated thyroid carcinoma (DTC) is the most common endocrine malignancy, accounting for about 3% of all malignancies (1). An increasing incidence of DTC has been reported in various parts of the world (1). Papillary carcinoma is the most common subtype of thyroid cancer accounting for 70-80% of all thyroid malignancies and is the variant that is causing the surge in the worldwide rate of DTC (2). However, the mortality from DTC is quite stable or shows a decreasing trend (3). As many as 50% of the patients develop cervical lymph node involvement, whereas 10-15% of the patients develop distant metastasis (4). The development of distant metastasis leads to a lower survival rate (5). 

 The common sites for metastasis are bone and lung, while the brain and liver are infrequently involved sites (6). Other sites such as skin and mediastinum have also been reported in the literature (6). We report a case of metastatic papillary thyroid cancer to the kidney in a 46-year-old male patient presenting with a left renal mass.

## Case Report

**Figure 1:**
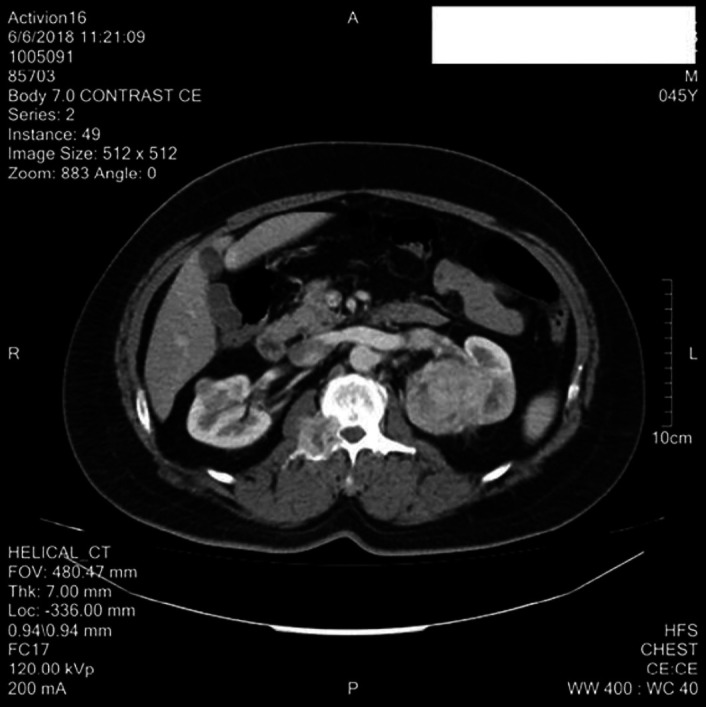
CT scan showing left renal mass along with the destruction of the right pedicle of the D12 vertebra

**Figure 2:**
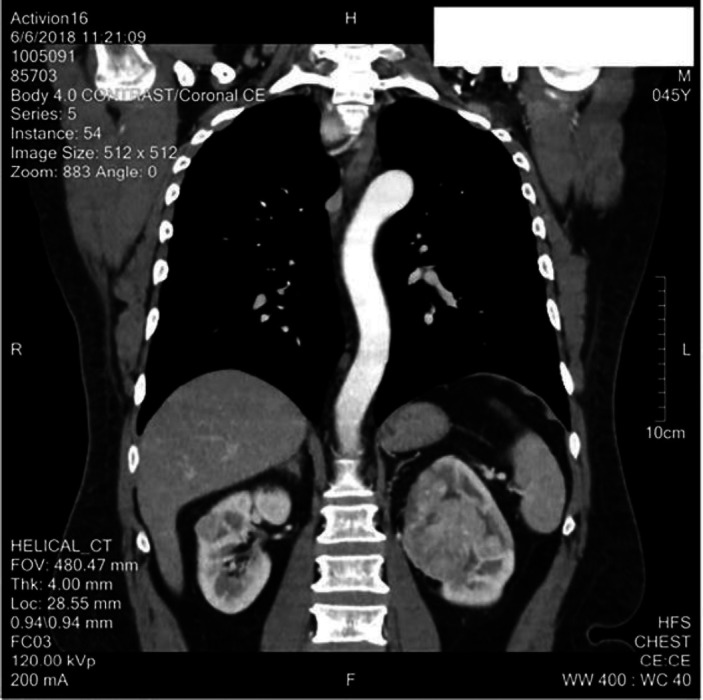
Coronal section of the CT showing renal left renal mass involving upper and mid pole

 A 46-year-old male patient presented with a complaint of abdominal pain for a few months. He had been taking over-the-counter pain medicine but the pain did not subside. He also complained of lethargy and dark coloured urine. He presented to an urologist, where on examination, he had a tender abdomen. A CT scan was performed that revealed a large heterogeneously enhancing soft tissue density mass that was involving the upper and mid pole of the left kidney (Figures 1 & 2). Since there were typical signs and symptoms and CT scan presentation, the workup for renal cell carcinoma was started and a CT chest and abdomen was done to rule out lung metastasis. 

 This CT scan showed no pulmonary consolidation or significant metastatic deposits. However, an enlarged right hilar lymph node was seen which measured approximately 1.7×1.6 cm. Tumour thrombus was noted in the left renal vein however inferior vena cava was spared. Destructive bony lesion involving the left glenoid labrum, right transverse process and pedicle of D12 vertebra were also noted (Figure 1).

**Figure 3:**
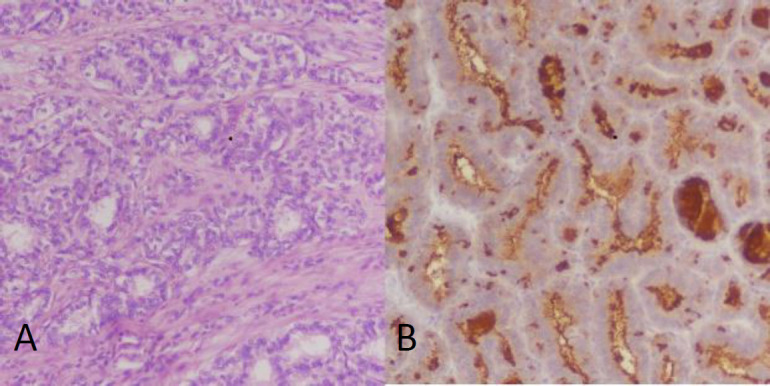
A renal mass with neoplastic lesion composed of colloid filled follicles lined with neoplastic cells displaying overlapping nuclei with grooves (**A**). Strong positive staining of thyroglobulin (**B**)

 Based on the findings a left-sided nephrectomy was performed and the sample was sent for histopathological examination. The mass specimen was 11×7×6 cm in diameter. On histapathological examination, neoplastic cells were seen, nuclear enlargement, clearing, overlapping and grooving. Immuno-histochemical studies by DAKO envision method was undertaken and CK 7, TTF-1 and thyroglobulin were found to be positive, while synaptophysin was found to be negative (Figure 3). Based on the morphological and histopatho-logical features, a definitive diagnosis of papillary thyroid carcinoma was established.

**Figure 4:**
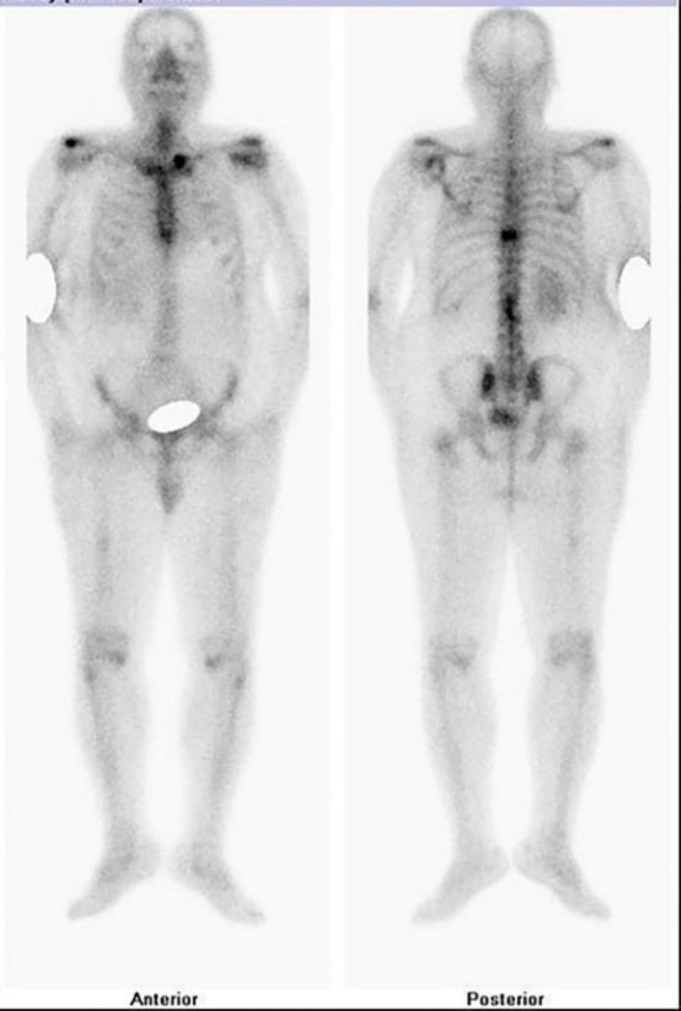
The whole-body ^99m^Tc-MDP bone scan of the patient revealing metastasis in the proximal end of the left clavicle, lateral border of the left scapula, D9 and L2 vertebrae and mid-shaft of right femur

 Subsequently, a thyroid ultrasound was done and it revealed a heterogeneous mass lesion completely replacing the right lobe of the thyroid gland. A few enlarged lymph nodes were also seen. A bone scan was done which showed metastasis involving the lateral border of the left scapula, the proximal end of the left clavicle, D9 and L2 vertebrae and the mid-shaft of the right femur (Figure 4).

**Figure 5:**
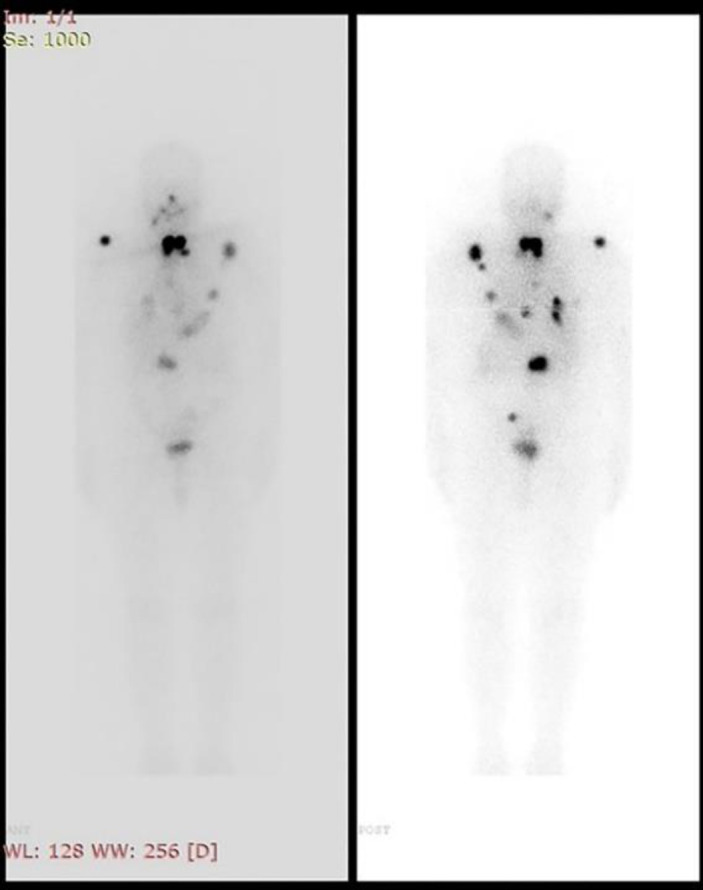
Post therapeutic whole body I-131 scan of the patient revealing multiple iodine avid metastases

 A total thyroidectomy was performed. Histopathology of the specimen revealed papillary thyroid carcinoma. The size of the tumor was 9.5×5×2 cm. Ten out of the 52 lymph nodes showed metastatic deposits. Thus the tumor was staged to be T3N1bM1. The largest lymph node measured 1.5×1.1 cm. His thyroglobulin level was 1250 ng/ml and the thyroglobulin antibody level was 16.91 IU/ml. Radioiodine therapy was administered post-operatively with a dose of 7.4 GBq (200 mCi) of I-131. A whole-body I-131 scan was done after 10 days which revealed residual functioning thyroid tissue in the thyroid bed along with multiple iodine avid lesions on the right posterior skull, bilateral shoulders & chest, mid and lower spine and left side of pelvis posteriorly (Figure 5).

**Figure 6:**
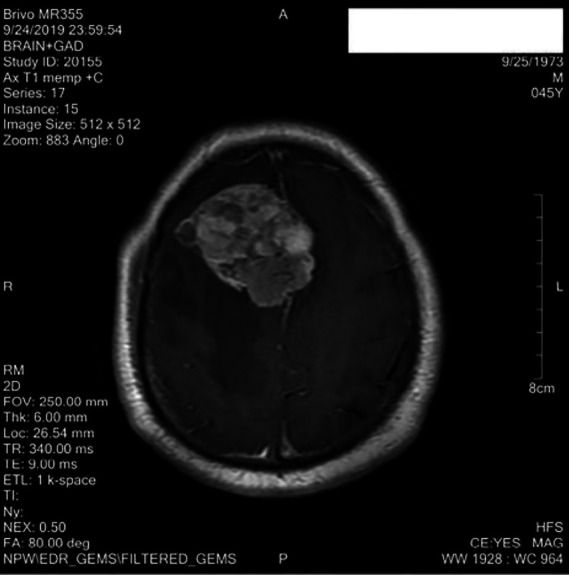
MRI revealing a large metastatic lesion in the right frontal lobe

 The patient was symptom-free for eleven months but subsequently developed headache and confusion. MRI was done on which showed a lobulated solid cum cystic mass in the right frontal parasagittal region with internal haemorrhages and surrounding moderate oedema (Figure 6.)

This mass measured 6.1×5.0×5.3 cm causing midline shift, compression over the right lateral ventricle and showing enhancement on post-contrast images. Keeping in view the history of metastatic thyroid cancer, a diagnosis of cerebral metastasis from the primary was made. Presently the patient is getting radiation therapy for cerebral metastasis. His latest thyroglobulin level is 1108 ng/ml and the thyroglobulin antibody level is 33.81 IU/ml. Further therapy with radioactive iodine is being considered.

## Discussion

 Any solid tumours may give rise to renal metastasis, however, most secondary lesions to the kidney occur from colorectal, lung, melanoma, breast, female pelvic tumours, esophagus and prostate (7). 

 Metastasis to the kidney of differentiated thyroid cancer is extremely uncommon with less than 30 cases being reported in the literature (8). In an autopsy series, only 6% of DTC metastasised to the kidney (9). Unlike in our case, in most cases of DTC metastasising to the kidney, thyroid cancer is discovered before the discovery of renal metastasis (8). Furthermore, in most cases, renal metastasis is asymptomatic. In most cases, patients had known thyroid tumours at the time of identification of renal metastases. In very rare instances, metastases to the kidney preceded the discovery of the primary thyroid neoplasm and were treated surgically as primary renal tumours. This is probably one of those rare cases in the literature where the renal metastasis had typical renal cell carcinoma like presentation and was treated as such prior to the diagnosis of ca thyroid.

 Falzarano et al (8) has reported two such cases where the renal metastasis mimicked renal cell carcinoma, in contrast to our case, both patients had a prior history of thyroid carcinoma. Similarly, Gezer et al (10) have also reported a case of thyroid cancer mimicking renal cell cancer. Yoon et al (6) in their article about unusual metastasis from thyroid cancer have also reported six cases that metastasized to the kidney. Thus thyroid carcinoma should also be considered in the differential diagnosis of a renal mass, even if no history of thyroid cancer is there.

## Conclusion

 Metastasis of thyroid cancer to kidneys is a rare entity, however it should be a part of the differential even in the absence of an obvious thyroid swelling.
